# A rapid nested polymerase chain reaction method to detect circulating cancer cells in breast cancer patients using multiple marker genes

**DOI:** 10.3892/ol.2014.2048

**Published:** 2014-04-08

**Authors:** LEI LIU, CHUNHU MA, QIAN XU, LUYANG CHENG, LIJUN XIAO, DAWEI XU, YAXIAN GAO, JIANPING WANG, HONGRU SONG

**Affiliations:** 1 Department of Immunology, Basic Medical Institute, Chengde Medical College, Chengde, Hebei 067000, P.R. China; 2 Clinical Skills Center, Chengde Medical College, Chengde, Hebei 067000, P.R. China; 3 Department of Central Laboratory, Basic Medical Institute, Chengde Medical College, Chengde, Hebei 067000, P.R. China

**Keywords:** breast cancer, tumor marker, circulating cancer cells, nested polymerase chain reaction

## Abstract

The aim of the present study was to develop a simple and rapid method for the detection of circulating cancer cells using multiple tumor markers and to investigate the clinical significance of circulating cancer cells in breast cancer patients. A novel rapid nested polymerase chain reaction (PCR) assay, with high sensitivity and specificity, was evaluated, which was considered to be suitable for clinical application. The rapid nested PCR method was used to detect the circulating cancer cells of 142 breast cancer patients, using a panel of marker genes (FAM83A, NPY1R and KRT19), which were identified by the Digital Gene Expression Displayer Tool of the National Cancer Institute-Cancer Genome Anatomy Project. In total, 79.6% of the 142 breast cancer patient blood samples were found to express at least one tumor marker. In addition, the number of positive markers was found to significantly correlate with the disease stage and presence of distant metastasis. Furthermore, positivity for more than one tumor marker appeared to predict a reduced survival time in breast cancer patients.

## Introduction

Breast cancer is the second most common type of cancer worldwide and undoubtedly the most common type of malignant disease in females. Despite the application of the American Joint Committee on Cancer tumor-node-metastasis system for staging and prognosis, ≤30% of node-negative patients ultimately develop recurrent disease (1). This may occur as a result of occult metastatic cells that are undetectable by current methods, which have spread via a lymphatic or hematogenous route. Therefore, it is of great clinical value to detect disseminated tumor cells using effective markers to supplement the staging method, prediction of metastasis and prognosis in breast cancer.

As breast cancer is highly heterogeneous and tumor cells continue to evolve genetically in response to host pressures, no single marker has been identified to be consistently and specifically expressed by all of the breast cancer cells. The positive detection rate of circulating cancer cells in breast cancer patients was only 43.9% when the single marker gene, cytokeratin 19, was employed (2). Therefore, a combination of multiple markers may be required to improve the sensitivity and specificity for the detection of circulating cancer cells. In addition, the number of circulating cancer cells is so small that they cannot be detected by conventional diagnostic methods, including imaging studies and assays for serum marker detection. However, it has been reported that the nest reverse transcription (RT)-polymerase chain reaction (PCR) is extremely sensitive and capable of detecting one breast cancer cell in 10^7^ cells, which is equivalent to four cells per 10 ml blood (3). However, such a technically demanding and time-consuming method is less suitable for clinical application.

In the present study, a simple and rapid nested PCR technique for the detection of circulating cancer cells in breast cancer patients is described; the method was based on designing two pairs of primers with marked differences in their annealing temperatures. In addition, a panel of markers was identified for the detection of circulating cancer cells in breast cancer patients by *in silico* analysis of the National Cancer Institute-Cancer Genome Anatomy Project database (http://cgap.nci.hih.gov/) (4). This rapid method was used to investigate clinical specimens obtained from breast cancer patients using the novel panel of marker genes. The panel of three marker genes was demonstrated to result in a significant improvement in the positive detection rate, which indicated that the positive expression of these markers correlates with the metastasis in, and prognosis of, breast cancer patients.

## Patients and methods

### Patients and samples

The present study was conducted using a total of 142 blood samples obtained from breast cancer patients, who were histopathologically and clinically diagnosed at the Affiliated Hospital of Chengde Medical College Cancer Center (Chengde, China) between November 2009 and December 2013. All patients provided written informed consent and the study was approved by the Ethics Review Committee of Chengde Medical College (Chengde, China). The patient age ranged from 21 to 82 years, with a mean age of 52 years. A total of 60 healthy female volunteers were also enrolled (median age, 49 years; range, 22–76 years). None of the patients received anti-hormonal treatment, chemotherapy, or radiotherapy prior to surgery. All data, including age, pathological type, tumor size, distant metastasis, clinical stage, estrogen receptor (ER), progesterone receptor (PgR), human epidermal growth factor receptor 2 and recurrence, were obtained from the clinical and pathological records.

Peripheral blood samples were obtained from superficial veins on the opposite side to the breast cancer by standard transcutaneous needle venipuncture and placed into a citrate sodium-containing tube. Two tubes were used to collect the blood, with 1 ml in the first tube and 5 ml in the second tube. The blood in the first tube was discarded as it may have been contaminated with epithelial cells picked up by the needle as it pierced the skin. However, the blood in the second tube was loaded on to a Ficoll-Hypaque layer (Gibco BRL, Carlsbad, CA, USA), and following density gradient centrifugation (Centrifuge HK-2C, Shenzhen Homk Telecommunication Technology Co., Ltd., Shenzhen, China) at 2,000 × g for 30 min at room temperature, the peripheral blood mononuclear cells (PBMCs) were collected. The PBMCs were washed twice using a sterile phosphate buffer solution. The cell pellets were subsequently snap frozen and stored at −80°C until RNA extraction.

### Identification of candidate marker genes

A large database of information regarding expressed sequence tags has been generated using cancer cell lines and is maintained on the cDNA Digital Gene Expression Displayer (developed by the National Cancer Institute-Cancer Genome Anatomy Project). This was used in the present study to identify the genes that were differentially expressed between breast cancer cells and leukocytes. The Digital Gene Expression Displayer program identified differentially expressed genes among 30,460 sequences in four breast cancer cDNA libraries and 21,036 sequences in five leukocyte cDNA libraries with the P filter set at 0.01. The differentially expressed genes were ranked by sequence odds ratio and the genes with the highest sequence odds ratios were selected as candidate marker genes for the RT-PCR assay.

### RNA preparation and cDNA synthesis

Total RNA was extracted using TRIzol reagent (Invitrogen Life Technologies, Carlsbad, CA, USA) according to the manufacturer’s instructions, treated with DNase I (Promega Corporation, Madison, WI, USA) and quantitated using ultraviolet spectrophotometry (UV2000; LabTech, Beijing, China). Subsequently, cDNA was synthesized from 2 μg total RNA using advantage reverse transcriptase (Clontech Laboratories, Inc., Mountain View, CA, USA). The integrity of the patients’ RNA samples and the fidelity of the cDNA synthesis were verified by a test amplification of GAPDH in a standard PCR reaction.

### Novel rapid nested RT-PCR assay

To detect the small number of cancer cells in the blood circulation, a novel, highly sensitive and rapid nested PCR technique was developed. Two pairs of primers with marked differences in their annealing temperatures (72 and 60°C for the outer and inner primers, respectively) were designed; the primer sequences are listed in Table I. The rapid nested PCR was performed using 2.5 μl of 10-fold diluted cDNA with a PCR mixture containing 0.2 μmol/l outer primers (100-fold dilution), 20 μmol/l inner primers, 0.2 mM deoxynucleotide triphosphate, 50 mM Tris-HCl, 10 mM KCl, 5 mM (NH_4_)_2_SO_4_, 2 mM MgCl_2_ and 0.75 units of *Taq* polymerase, in a total volume of 25 μl. The PCR conditions used were as follows: 95°C for 5 min; 30 cycles at 95°C for 20 sec and 72°C for 1 min; 20 cycles at 95°C for 10 sec, 60°C for 20 sec and 72°C for 10 sec; and a final extension at 72°C for 7 min.

To evaluate the novel rapid nested PCR technique, the traditional nested PCR was also performed using 1 μl of 20-fold diluted cDNA with a PCR mixture containing 20 μmol/l outer primers, 0.2 mM deoxynucleotide triphosphate, 50 mM Tris-HCl, 10 mM KCl, 5 mM (NH_4_)_2_SO_4_, 2 mM MgCl_2_ and 0.75 units of *Taq* polymerase in a total volume of 25 μl. The PCR conditions used were as follows: 30 Cycles at 95°C for 20 sec and 72°C for 50 sec; and a final extension at 72°C for 7 min. Next, 2 μl of the first PCR product (1:100) was used as a template for the following round of PCR and the conditions were as follows: 30 Cycles at 95°C for 20 sec, 60°C for 20 sec and 72°C for 20 sec; and a final extension at 72°C for 7 min.

The positive and negative controls were included in each run and all precautions to prevent cross-contamination were observed. Visualization of the target bands was performed using a 1.0% agarose gel with ethidium bromide staining to determine the expression of the mRNA transcripts.

### Follow-up

A follow-up study of the 142 breast cancer patients was conducted by telephone interview, between November 2009 and December 2013 with additional verification of their clinical records. Chest X-rays and mammographs were examined biannually, and liver ultrasounds and bone scans were examined annually.

### Statistical analysis

Statistical analyses were conducted using SPSS 17.0 statistical package (SPSS, Inc., Chicago, IL, USA) and the χ^2^ test was performed to determine the correlation between the marker expression status and clinicopathological features. The survival distributions were investigated using Kaplan-Meier methods and the log-rank test was used to assess the statistical significance of differences in overall survival between the different groups. P<0.05 was considered to indicate a statistically significant difference.

## Results

### Marker genes for detecting circulating breast cancer cells

The *in silico* Digital Gene Expression Displayer program search of the National Cancer Institute-Cancer Genome Anatomy Project database yielded 23 overexpressed genes with a sequence odds ratio of >16 between the breast cancer and leukocyte cDNA libraries. The nested PCR was used to further verify the candidate genes in the peripheral blood samples of the 43 breast cancer patients and 20 healthy control subjects, whereby three marker genes, including FAM83A (NM_032899.4), NPY1R (NM_000909.5) and KRT19 (NM_002276), were identified as the novel panel of markers. Of the 43 breast cancer patients, 14 patients exhibited NPY1R expression and 16 patients expressed FAM83A, however, these two marker genes were undetectable in the peripheral blood of the 20 healthy control subjects. Furthermore, the marker gene, KRT19, showed positive expression in 21 breast cancer patient samples, however, only one patient expressed the gene in the healthy control group.

### Evaluation of the rapid nested PCR assay

**Figure 1:**
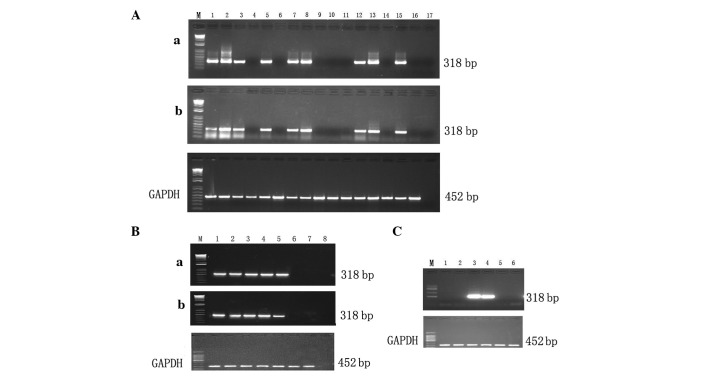
Evaluation of the rapid nested polymerase chain reaction (PCR). (A) The same detection rate was achieved using the (a) novel rapid and the (b) traditional nested PCR assays. Lanes 1–15, breast cancer patients; lane 16, healthy donor; and lane 17, negative control. (B) Sensitivity of the (a) novel rapid and the (b) traditional nested PCR assays. Lanes 1–7, 10-fold serial dilution of MCF-7 using peripheral blood mononuclear cells obtained from healthy donors (Lane 1, 10^2^; lane, 2 10^3^; lane 3, 10^4^; lane 4, 10^5^; lane 5, 10^6^; lane 6, 10^7^; and lane 7, 10^8^ cells); and lane 8, negative control. (C) Amplification using outer and inner primers. Lanes 1 and 2, outer primers only; lanes 3 and 4, outer and inner primers; and lanes 5 and 6, inner primers only.

Using the novel rapid and traditional nested PCR techniques to detect the circulating cancer cells of 43 breast cancer patients, the same 16 samples exhibited FAM83A expression in the two assays (Fig. 1A). The relative sensitivity was determined by a 10-fold serial dilution of the breast cancer MCF-7 cell line using PBMCs obtained from the healthy donors. The breast cancer MCF-7 cell line was detected at a dilution of 10^−6^ using the rapid nested PCR and the traditional nested PCR techniques, indicating that the two assays exhibit the same sensitivity (Fig. 1B). The target bands were produced with outer and inner primers, while no target bands were amplified using only outer or inner primers (Fig. 1C). The amplification times were 65 and 89 min for the rapid and traditional nested PCR assays, respectively, shortening the total duration to 24 min.

### Enhancement of positive detection rate with multiple markers

**Figure 2:**
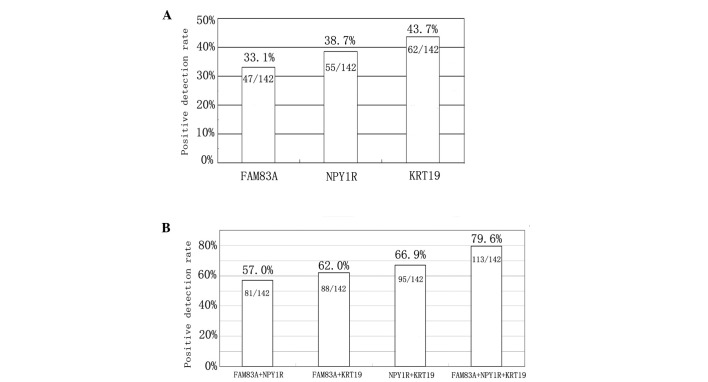
Analysis of positive detection rates with a multimarker gene panel. The positive detection rate for (A) the three individual marker genes. (B) The positive detection rate increased with the panel of markers.

The three candidate markers, FAM83A, NPY1R and KRT19, were investigated further in a large cohort consisting of 142 breast cancer patients and 60 healthy controls, using the novel rapid nested PCR assay. As shown in Fig. 2A, the positive detection rate of circulating cancer cells in breast cancer patients was 33.1 (47/142), 38.7 (55/142) and 43.7% (62/142) for the FAM83A, NPY1R and KRT19 genes, respectively. The FAM83A and NPY1R transcripts were undetectable in the PBMC samples of the 60 healthy controls, whereas the KRT19 marker was detected in three of the healthy samples. The fraction of positives among all of the patients are indicated in Fig. 2 and ≤79.6% (113/142) of the breast cancer patient blood samples were found to be positive for at least one of the markers. The novel panel of the three gene markers, FAM83A, NPY1R and KRT19, was found to be significantly superior to the individual markers or any combination of two markers. These results identified that using multiple markers improves the positive detection rate (Fig. 2B).

### Tumor marker detection and patient characteristics

The statistical analysis was performed to determine the correlation between marker expression frequency and clinicopathological variables. With regard to the clinical stage, the detection rate of FAM83A and NPY1R, as well as the panel of markers, was significantly higher in patients with stage III or IV breast cancer when compared with stage I or II patients (P<0.05). The detection rate of the three marker genes, or at least one of the three markers, was found to correlate with the occurrence of distant metastasis (P<0.05), which indicated the benefit of the panel as a predictive peripheral blood marker for metastasis in breast cancer. In addition, the expression rate of NPY1R was significantly higher in ER−, PgR− or HER2/*neu*-positive patients when compared with ER−, PgR− or HER2/*neu*-negative patients (P<0.05). However, no statistically significant correlation was identified between marker detection and tumor size, pathology type or patient age (P>0.05; Table II).

### Correlation between tumor marker gene detection and disease progression

**Figure 3:**
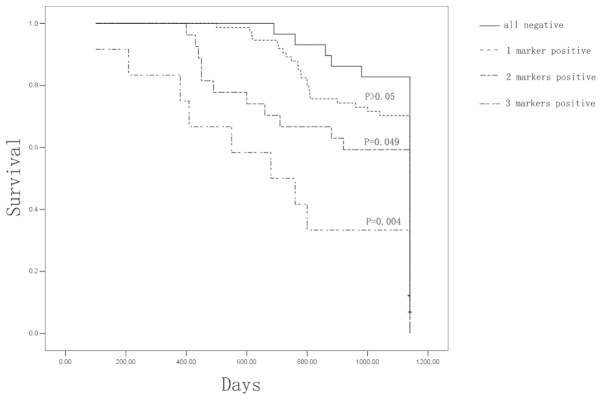
Kaplan-Meier survival analysis for breast cancer patients, grouped according to the number of tumor markers detected in the peripheral blood.

To investigate the correlation between the detection of circulating cancer cells and the clinical outcome of breast cancer patients, a follow-up study was performed for 38 months in 142 patients following surgical removal of the tumor mass. The survival rate was 82.8% (24/29) for the all-negative patients (those that exhibited no tumor markers), 70.3% (52/74) for patients that were positive for a single tumor marker, 59.3% (16/27) for patients that were positive for two tumor markers and 33.3% (4/12) for patients that were positive for three tumor markers. In addition, the Kaplan-Meier analysis and log-rank test indicated no difference in the overall survival rate between the all-negative and single-positive marker patients (P>0.05). However, patients that were positive for more than one tumor marker exhibited a significant overall disadvantage when compared with the all-negative patients (P<0.05; Fig. 3).

## Discussion

The detection of circulating cancer cells is a promising and powerful tool for cancer diagnosis and disease monitoring (5). However, due to the heterogeneous expression of individual markers in tumor lesions within individual patients or among patients, the predictive power of a single marker is relatively limited. In the present study, *in sillico* analysis was performed to identify a panel of marker genes for the detection of breast cancer cells dispersed in the circulation. Based on the experimental results of the current study, the National Cancer Institute-Cancer Genome Anatomy Project database and Digital Gene Expression Displayer program were considered to be useful tools for establishing the genes that were expressed between the two pools of samples. However, this is only reliable when a sufficient quantity of expressed sequence tag libraries for the tissue of interest are archived in the database (6).

The differentially expressed genes can be further developed into marker genes for diagnostic or prognostic purpose by experimental verification procedures such as RT-PCR. The favorable marker must exhibit a high level of expression in breast cancer tissues, however, no or low expression in healthy PBMCs. In addition, the current study identified a novel panel of marker genes (FAM83A, NPY1R and KRT19) for the detection of cancer cells in the peripheral blood of breast cancer patients. KRT19 is a characteristic marker of epithelial cells (7), whereas FAM83A is a novel biomarker for detection of the peripheral blood, which was identified as a tumor-specific gene in our previous study (8). Furthermore, the FAM83A mRNA transcript was expressed in 21 of 40 lung cancer samples (52.5%), 24 of 50 breast cancer samples (48.0%), four of 12 colon cancer samples (33.3%) and three of 10 gastric cancer tissues (30.0%), however, was not detected in the 16 healthy tissues. The overall positive rate of FAM83 gene expression in the peripheral blood was 34.3% (24/70) (8).

NPY1R was the first NPY receptor subtype to be cloned and characterized. Previous studies revealed that normal breast tissue expresses the Y2R subtype, whereas 85% of human breast carcinoma express NPY1R (9). It has been proposed that an interaction between estrogen, and NPY and its receptors illustrates the concerted action of estrogen and progesterone on increasing NPY levels, which results in an associated increase in the release of luteinizing hormone (10). In the current study, the expression of the marker gene, NPY1R, in peripheral blood was found to correlate with the expression of ER and PgR, whereby the expression rate of NPY1R was significantly higher in the ER− and PgR-positive group compared with that in the ER− and PgR-negative group. Furthermore, the results indicated that estrogen is important in the upregulation of NPY1R, which in turn regulates estrogen-induced cell proliferation in breast cancer cells (11).

The number of circulating cancer cells is so marginal that they cannot be detected using conventional diagnostic methods. Various techniques have been developed to enrich certain types of cells from the blood and to characterize these cells using nested RT-PCR assays, which is extremely sensitive and capable of detecting one breast cancer cell in 10^7^ cells; however, this two-step, time-consuming method is less suitable for clinical application. To overcome this problem, the current study developed a simple and rapid nested PCR assay, which is less time-consuming and, therefore, more readily applicable to the clinical investigation of relatively large sample numbers in clinical practice. Firstly, two pairs of primers were designed, that had marked differences in their annealing temperatures, (72 and 60°C for outer and inner primers, respectively). The two steps of the nested PCR reaction were performed in one tube. The first step consisted of heating the reaction to 95°C, causing the denaturation of the DNA template. Next, the reaction temperature was lowered to 72°C to allow annealing of the outer primers to the single-stranded DNA template. Following 30 cycles of amplification, a 100-fold dilution of the outer primers (0.2 μmol) was depleted to achieve a large amount of product in the tube, which was denatured upon reheating the reaction to 95°C. The reaction temperature was lowered to 60°C to allow the annealing of the inner primers to the single-stranded product template. It was verified that the target bands were produced with the outer and inner primers; however, none of the target bands were amplified with only the outer or inner primers.

The duration of the reaction using the improved method was ~1 hr; the volume of the PCR mix was 25 μl and the PCR reagents were added using a one-step method. The amplification results indicated that the PCR factors used were effective and that the enzymatic activity of the *Taq* DNA polymerase had been preserved. The specificity and sensitivity of the amplification were the same as those of the traditional method, and the cost of the test was reduced proportionally due to the decreased use of reagents.

In conclusion, the current study developed a novel rapid nested PCR assay to detect circulating cancer cells in the blood of breast cancer patients using a novel panel of marker genes, FAM83A, NPY1R and KRT19. This assay may present a useful tool for the prediction of cancer metastasis in breast cancer patients and for the evaluation of their prognosis.

## Figures and Tables

**Table I:** Primer sequences used in the polymerase chain reaction for detecting the three marker genes.

Gene	Primer sequence	Annealing temperature, °C	Product, bp
FAM83A			318
Outer		72	
Forward	5′-CGCCACTGTGTACTTCCAGACCGTCAAGC-3′		
Reverse	5′-CCTCGGCGGTTCTGCTCATGCTCCACTC-3′		
Inner		60	
Forward	5′-GTGGGGTGTTCGTTTGTG-3′		
Reverse	5′-GCTTGGAGGAGGCGTAG-3′		
NPY1R			372
Outer		72	
Forward	5′-GCGTTCCAAAATGTAACACTTGATGCGTACA-3′		
Reverse	5′-CATCTGTGTGCATCGTGGACATGGCTATTGT-3′		
Inner		60	
Forward	5′-CACTCTTCTCTTGGTGCTG-3′		
Reverse	5′-GTTTTTGTTCAGGAACCCA-3′		
KRT19			393
Outer		72	
Forward	5′-AAGCTAACCATGCAGAACCTCAACGACCGC-3′		
Reverse	5′-TTTTATTGGCAGGTCAGGAGAAGAGCC-3′		
Inner		60	
Forward	5′-CAGCCACTACTACACGACC-3′		
Reverse	5′-ACCTCATATTGGCTTCGCA-3′		
GAPDH		60	452
Forward	5′-ACCACAGTCCATGCCATC-3′		
Reverse	5′-TCCACCACCCTGTTGCTGTA-3′		

**Table II:** Characteristics and tumor marker expression in the circulating cancer cells of breast cancer patients.

Characteristic	n	FAM83A, n (%)	NPY1R, n (%)	KRT19, n (%)	Positive rate^a^, n (%)
Age, years					
<50	56	17 (30.4)	21 (37.5)	25 (44.6)	46 (82.1)
≥50	86	30 (34.9)	34 (39.5)	37 (43.0)	67 (77.9)
Pathology					
Invasive ductal carcinoma	98	33 (33.7)	38 (38.8)	43 (43.9)	78 (79.6)
Simple cancer	7	2 (28.6)	3 (42.9)	3 (42.9)	6 (85.7)
Eczematous cancer	5	2 (40.0)	2 (40.0)	2 (40.0)	4 (80.0)
Medullary carcinoma	19	6 (31.6)	7 (36.8)	8 (42.1)	15 (78.9)
Invasive lobular carcinoma	13	4 (30.8)	5 (38.5)	6 (46.2)	10 (76.9)
Tumor size, cm					
≤2	75	25 (33.3)	29 (38.7)	32 (42.7)	59 (78.7)
>2	67	22 (32.8)	26 (38.8)	30 (44.8)	54 (80.6)
Clinical stage					
I, II	89	22 (24.7)	27 (30.3)	36 (40.4)	64 (71.9)
III, IV	53	25 (47.2)^b^	28 (52.8)^b^	26 (49.1)	49 (92.5)^b^
Distant metastases					
No	121	36 (29.8)	42 (34.7)	48 (39.7)	93 (76.9)
Yes	21	11 (52.4)^b^	13 (61.9)^b^	14 (66.7)^b^	20 (95.2)^b^
Estrogen receptor status					
Positive	101	34 (33.7)	44 (43.6)^b^	45 (44.6)	82 (81.2)
Negative	41	13 (31.7)	11 (26.8)	17 (41.5)	31 (75.6)
Progesterone receptor status					
Positive	89	30 (33.7)	40 (44.9)^b^	41 (46.1)	71 (79.8)
Negative	53	17 (32.1)	15 (28.3)	21 (39.6)	42 (79.2)
HER-2/*neu* receptor status					
Positive	50	19 (38.0)	25 (50.0)^b^	23 (46.0)	40 (80.0)
Negative	92	28 (30.4)	30 (32.6)	39 (42.4)	73 (79.3)

a At least one marker gene was detected.

b P<0.05 vs. the other group.
